# Supplementary material for: Are carbon emissions trading and green financial instruments synergistic? -Comprehensive quantitative research based on content analysis
Source: PLoS One. 2024 Mar 7;19(3):e0298601. doi: 10.1371/journal.pone.0298601 (PMC10919601; doi:10.1371/journal.pone.0298601)
Supplement: S1 Data — (ZIP) [file pone.0298601.s001.zip › Data/地级市Z_F工作报告.zip!/║╙─╧/░▓╤⌠╩╨/└┤╫╘╣1⁄2╓┌║┼í╛┬φ┐╦╩2╛▌═°í┐_2013.docx]

各位代表:

现在，我代表市人民政府，向大会报告政府工作，请予审议，并请各位政协委员提出意见。

折叠编辑本段2012年工作回顾

一年来，我们突出抓了以下重点工作:

(一)抓帮扶，促增长，科学有效应对危机。针对不同阶段经济运行的突出矛盾和特点，围绕保企业、扩内需、拓市场等关键环节，及时制定实施了一系列有针对性的政策措施，有效扭转了经济下行态势。积极帮扶企业。先后出台促进困难行业转型解困等8个专题工作方案，建立百名县级干部分包联系百户重点企业制度，成立钢铁、焦化、装备制造等8个帮扶重点行业、88个帮扶重点企业工作组，积极开展产销对接、银企对接活动，帮扶困难企业开拓市场、筹措资金、减压降负、共渡难关。着力扩大投资。成立24个重点工作领导小组，谋划实施"八个方面十大工程"和"8415"推进工程。安化PET、国药集团安阳医药物流中心、河南东泰医药产业园等项目开工建设。林州重机煤机、凯瑞数码双面单层可录光盘、汇丰管业无缝钢管等项目全面竣工。大通微电子新产品下线。滑县盈德气体、内黄高档陶瓷、汤阴阳光油脂植物蛋白及食用油等项目加快建设。167个省、市重点项目完成投资288.7亿元，为年度目标的124%。努力促进消费。进一步完善农产品流通体系，深入推进万村千乡市场工程，新建改建农家店3155家。"放心早餐"工程继续巩固。强化价格监测调控，全市居民消费价格指数低于全国、全省平均水平。保障要素供应。着力破解融资难题，激励金融机构加大信贷投放力度，全年实际新增贷款82.96亿元，同比增长14%。加强煤电油气运调节，强化土地、人才、劳动力保障供应，较好保障了重点项目建设和正常生产生活需要。

(二)抓调整，促转型，加快发展方式转变。一手抓增长，一手促转型，经济发展质量和资源利用水平进一步提升。加快产业集聚区发展。全市产业集聚区完成固定资产投资500.5亿元，规模以上工业企业主营业务收入1192.4亿元。装备制造、食品医药等行业集群化发展态势加快。安阳县、汤阴县等产业集聚区规划调整获批。内黄县被授予中原陶瓷产业基地称号。安阳新区、中原高新技术产业开发区正式成立。林州红旗渠经济开发区晋升为国家级经济技术开发区。优化产业产品结构。大力实施"传统产业升级行动计划"和"新兴产业倍增行动计划"，109个工业振兴工程重点项目全部开工，完成投资251亿元。安钢4800立方米高炉、安化乙二醇、新普钢铁棒材升级等56个高新项目竣工。豫北金铅、岷山有色等技改工程完成。远大集团可建项目落户安阳。与中建材、日本松下能源社签订合作协议。我市被列入国家中部老工业基地调整改造市。积极推进光伏应用示范工程，建设光伏发电项目11个。全市13家企业入围省"百强"、"百高"企业。大力发展第三产业。成功举办第四届中国·安阳国际航空运动旅游节、2012中国·安阳殷商文化旅游节。积极实施十大文化旅游工程，殷墟国家考古遗址公园、中国文字博物馆续建等项目加快推进。我市被批准为国家通用航空产业园区试点。市会展中心、安阳迎宾馆建成投用。现代市场体系建设积极推进。市商务中心区获批，3个特色商业区规划通过省专家评审。安阳路港国际物流园、海尔虚实网服务园等成功签约。着力提高自主创新能力。新增2家省工程技术研究中心，4家企业入选省高新技术特色产业基地骨干企业，2家企业成为省创新型企业。高新技术产业增加值30.77亿元，同比增长19%。创建中国驰名商标3个，实现零突破。鑫盛机床精密数控车床、安阳锻压数控锤等13个项目入选国家火炬计划。节能减排成效显著，预计万元生产总值能耗下降6%，主要污染物减排目标圆满完成，环境质量逐步改善。

(三)抓引领，促协调，构建现代城镇体系。突出新型城镇化引领，努力形成城乡一体化发展新格局。加快完善城市基础设施。坚持中心城市带动，加快组团发展，水冶镇、汤阴县城、内黄县城被列入省第一批城市组团名单。安阳新区路网、学校、医院、水系景观等配套工程加快推进。安汤新城总体规划和路网规划已经完成。中华路北段建成投用，解放大道全线拓宽和一中路口、三角湖路口等改造完成。文峰中路商业街、京港澳高速公路南出入口等道路亮化到位。旧城和"城中村"改造稳步推进。我市被评为全省精细化管理优秀城市。着力构建现代交通体系。林长高速公路、石武高铁等建成通车。107国道升级改造、晋豫鲁铁路通道进展顺利。豫东北机场选址确定。大力推进新型农村社区建设。出台了《关于加快推进新型农村社区建设的实施意见》等文件，投入资金133.5亿元，基本建成新型农村社区16个。17个乡镇入围全省百强乡镇，水冶镇连续8年保持综合实力第一名。

(四)抓基础，促增收，推动农村经济全面发展。认真落实强农惠农政策，保持了农业农村发展的好形势。粮食总产349.6万吨，同比增长3.4%，连续10年增产。滑县荣获全国粮食生产先进县"十连冠"称号。深入推进农业结构调整。新增温棚设施高效农业4.1万亩、立体高效农业5.3万亩。新建扩建高效农业园区47个、规模以上龙头企业30家、大中型规模养殖场210个。新发展农民专业合作社392家。全面加强农业基础设施建设。34座小型病险水库除险加固工程竣工，5条河流治理工程完成。内黄县、滑县引黄灌区平原水库开工建设。南水北调安阳配套工程全面启动。安阳河于曹闸主体完工。连续19年夺得省"红旗渠精神杯"。切实改善农村生产生活条件。新建大中型沼气池64座，新增沼气用户1.5万户。解决了44.2万人饮水安全问题。新改建农村公路246公里。完成50个贫困村整村推进任务，实现5.45万农村人口脱贫。扎实推进林业生态建设。全年营造林14.4万亩，"两岭"和五大森林公园造林开发1.5万亩。全国绿化模范城市创建工作扎实推进。汤河湿地公园批准为国家湿地公园。

(五)抓开放，促改革，增强经济发展动力活力。把开放改革作为应对危机、促进转型、实现科学发展的根本动力，持续集聚发展正能量。强力推进开放招商。组织开展了百名粤商话安阳、天津滨海新区洽谈会等一系列重大招商活动。组织44支小分队，分赴上海、天津、昆山等地开展驻地招商。凤竹高端纺织基地、深圳华强产业园、北京贯辰通用航空基地等落地开工。中国五矿安阳产业园、柏庄义乌中原纺织服装城、国际汽博产业园、精密铸造产业园、豫科龙盛空冷设备工业园等成功签约。全年引进省外资金374.6亿元，同比增长23.9%;实际利用外资3.16亿美元，同比增长22%。坚持深化改革创新。非公有制经济不断壮大，占全市生产总值比重达60%。水泥、煤焦化等企业战略重组取得积极进展。6家企业被确定为省重点上市后备企业。我市被评为全省文化体制改革先进地区。科技、教育、医药卫生、事业单位等改革顺利推进。

(六)抓民生，促和谐，营造良好社会发展环境。在经济困难的形势下，始终坚持民生优先，财政用于"十项民生工程"支出63.2亿元，46件实事基本完成。全力做好就业工作。新增城镇就业8.3万人，再就业3.2万人，其中，困难群体再就业1万人。农村劳动力转移就业178万人。不断完善社会保障体系。新型城镇居民养老保险实现全覆盖，企业退休人员养老金、城乡低保和优抚对象补助水平逐步提高。新农合参合率连续三年稳定在99%以上。新开工保障性住房2.28万套，竣工1.12万套。全面发展社会事业。义务教育均衡发展，城乡薄弱学校办学条件不断改善，新建、改扩建幼儿园174所，职业教育、高等教育发展实现新突破。公共图书馆、文化馆全部免费开放。新建1117个农家书屋。国家基本药物制度实现基层全覆盖。成功举办第二届全民健身大会。人事人才工作进一步加强。低生育水平持续稳定。积极开展食品安全"四大放心工程"，食品药品监管全面加强。安全生产形势稳定好转。稳妥推进非法集资案件处置，信访态势保持平稳。社会治安综合防控体系进一步完善。加强社会管理和创新，社会大局总体稳定。

精神文明和民主法治不断加强。自觉接受人大法律监督、工作监督和政协民主监督，认真办理人大代表议案、建议和政协委员提案。深入推进反腐倡廉建设，不断加强行政效能监察。坚持军民融合式发展，军政军民团结进一步巩固。老区建设不断加强。依法保护妇女儿童合法权益。社会福利、慈善事业和老龄、红十字、残疾人、关心下一代工作健康发展。统计、审计、史志、档案、气象、地震、邮政、发展研究、外事侨务、民族宗教等工作进一步加强。

各位代表!

刚刚过去的一年，国际金融危机的影响进一步扩大，世界经济持续低迷，国内经济增速放缓，我市经济社会发展遇到了前所未有的困难和挑战。一年来，我们在困难中经受了考验，在实干中凝聚了力量，在发展中积累了经验，对加快发展有了更深刻的理解和体会:一是必须坚持产业转型发展方向。更加注重经济转型，引导产业向优势集聚，产品向高端发展。二是必须坚持新型城镇化引领。通过加快新型农村社区、城市新区、城市组团的规划建设，增强扩大内需的持久动力，为经济发展提供强大引擎。三是必须坚持开放招商根本途径。积极承接产业转移，吸引资金、技术、人才等要素向我市集聚，实现借力发展。四是必须坚持执政为民根本目的。把维护好、实现好、发展好广大人民群众的根本利益作为一切工作的出发点和落脚点，让发展成果更多地惠及全市人民。五是必须坚持务实重干作风保障。统一思想认识，突出实干实效，强化务实发展，努力营造团结协作、克难攻坚、竞相发展的良好氛围。

各位代表!

在复杂严峻的形势下取得这些成绩，来之不易。这是市委正确领导的结果，是市人大、市政协大力支持和监督的结果，是全市人民和衷共济、团结奋斗的结果。在此，我代表市人民政府，向奋斗在全市各条战线上的广大工人、农民、干部、知识分子、解放军指战员、武警官兵、政法干警以及各界人士，致以崇高的敬意!向所有关心、支持我市现代化建设的港澳台同胞、海外侨胞、国际友人，表示衷心的感谢!

总结成绩的同时，我们也清醒地认识到，由于我市钢铁、化工等支柱行业受到严重冲击，加之长期积累的体制性、结构性矛盾叠加凸显，去年年初确定的全市生产总值、公共财政预算收入、规模以上工业增加值增速三项指标，经市人大第28次常委会调整后才基本完成。与此同时，经济社会发展中还存在一些不容忽视的矛盾和问题:一是产业结构不合理、终端产品少、高新技术产业占比偏低、综合竞争力不强等问题在危机中愈加凸显，部分行业和企业生产经营仍然困难。二是城镇化水平较低，老城区基础设施和公共服务设施还不够完善，城乡统筹步伐有待进一步加快。三是市本级财源建设不足，部分领域改革改制不到位，一些项目推进力度不够。四是公共服务水平仍然较低，一些涉及群众切身利益的就业、就学、就医、住房等问题尚未得到很好解决。特别是非法集资对金融秩序和社会稳定的影响仍然较大。五是一些干部的知识结构、领导方式和工作方法与新形势、新任务的要求不相适应。个别部门和单位，办事拖拉，效率不高，为企业、为基层服务的意识不强，发展环境有待进一步优化。对此，我们一定高度重视，认真解决。

折叠编辑本段2013年总体要求和奋斗目标

今年是全面贯彻落实党的十八大精神的开局之年，是实施"十二五"规划承前启后的关键一年，也是为全面建成小康社会奠定坚实基础的重要一年，做好今年工作意义重大。今年，我市面临的外部环境更为复杂严峻。世界经济已由危机前的快速发展期进入深度转型调整期，经济低迷成为全球经济新常态。我国经济已进入实质转型期和个位数增长阶段，需求不足短期内难以改观，国内产能过剩矛盾短期内难以明显缓解，特别是受国家房地产调控政策影响，钢材、水泥等原材料需求严重不足。区域竞争更加激烈，在争取资金、政策、项目、人才等方面，各地千帆竞发、百舸争流，稍有懈怠就会陷于被动。同时也要看到，我市发展仍处在重要战略机遇期，经济总体向好的基本面没有改变。国内外产业加速向我国中西部地区转移的态势没有改变，我市仍处于开放招商、承接产业转移的"黄金期"。中央"稳中求进"的总基调没有改变，《中原经济区规划》正式颁布，为我们加快发展提供了难得的战略机遇和政策环境。我市新型城镇化、新型工业化加速推进趋势没有改变，竞争优势不断增强，发展后劲持续积蓄，全市上下干事创业的气势更加高涨，为我市经济增长提供了重要支撑。综合来看，尽管形势严峻，但有利条件多于不利因素，机遇大于挑战。我市已进入爬坡过坎、攻坚转型的关键期，我们既要正视困难和问题，始终保持清醒忧患，做好应对更大困难和挑战的准备，更要看到机遇和优势，在应对挑战、化解风险、克服困难、解决问题中坚定信心，因势利导，科学决策，推动经济社会又好又快发展。

今年政府工作的总体要求是:深入学习贯彻落实党的十八大精神，坚持以邓小平理论、"三个代表"重要思想、科学发展观为指导，抓住中原经济区建设的战略机遇，按照"富民强市、晋位争先"的总体要求，围绕"经济转型、方式转变"这一主线，实施"四大战略"，以提高经济增长质量和效益为重点，稳中求进，持续提升，开拓创新，扎实开局，保持"三势"，迈出全面建成小康社会和中原经济区区域性中心强市建设的新步伐。

今年经济社会发展主要目标是:生产总值增长8%;规模以上工业增加值增长11%;公共财政预算收入增长10%;全社会固定资产投资增长18%;社会消费品零售总额增长16%;外贸进出口增长15%，实际利用外资增长10%;城镇居民人均可支配收入、农民人均纯收入均增长9%左右;城镇新增就业6.5万人以上;城镇登记失业率控制在4.5%以内;人口自然增长率控制在6.45‰以内;居民消费价格涨幅控制在3.5%左右;节能减排完成省定目标。

实现上述要求和目标，必须坚持"总要求"，牢牢把握主题主线，富民强市、晋位争先。必须转变"立足点"，把推动发展的立足点切实转到提高质量和效益上来。必须突出"四着力"，着力以体制改革和机制创新培育发展新活力，以创新驱动和新型城镇化引领培育发展新动力，以载体完善提升和优化结构升级培育现代产业发展新体系，以强化基础支撑和优化发展环境培育发展新优势。必须抓好"三重点"，扩大对外开放、承接产业转移，"一举求多效"，强化新型城镇化引领、促进"三化"协调发展，"一发动全身"，优化软硬环境、壮大发展基础，"一优带百通"，以重点突破带动全局。必须推进"四转型"，由老工业基地向新型工业城市、由文化旅游资源大市向文化旅游名城、由区域性交通枢纽向区域性交通物流商贸中心、由重要农产品生产基地向新型农业现代示范区转型。必须加快"五转变"，在经济结构、发展动力、工业结构、企业战略、产品结构上实现转变。

折叠编辑本段2013年工作安排

完成今年各项目标任务，必须统筹兼顾、突出重点，加快转型、完善提升，不断研究制定应对困难更直接、更有力、更见效的政策措施，努力保持经济持续健康发展。今年，要着重抓好九项工作:

(一)着力加快产业产品结构调整，不断推进老工业基地向新型工业城市转型。大力推进产业结构调整和集聚集群发展，不断提高终端、高端和高附加值产品比重，加快转型升级，推动老工业基地振兴。加快传统产业结构转型。对传统产业进行调整和改造，延伸产业链条，引进高新技术，提高竞争力。钢铁业方面，支持以安钢集团和沙钢永兴为龙头，加快发展钢材精深加工，逐步形成全省精品钢生产基地。煤化工业方面，以河南煤化集团安化公司和铜冶四大焦化企业为依托，拉长煤化工产业链条。装备制造业方面，打造煤矿大型成套装备、数控机床、数控锻压设备、动力与轻化工装备等4条优势产业链，引导产品向高端装备制造和精密加工方向转移。食品医药业方面，大力推动健丰、旺旺、永达、益海嘉里、科伦、东泰、九州等优势企业快速扩张，培育国家知名品牌。纺织业方面，发挥安阳针织内衣产业优势，建设纺织制造、加工、商贸集聚区，促进产业集群式发展。加快新兴产业培育。重点发展新能源、生物医药、节能环保、新材料、高端装备制造等高成长性、战略性产业。电子信息产业方面，大力发展手机、电脑、软件、光盘、动力电池和物联网服务等产业，全面提升产业配套能力。新能源产业方面，推动光伏示范应用，向光伏装备制造、光伏建筑、光伏并网电站等产业延伸。加快产品结构转型。推进产品结构优化，着力解决产业链条短、产品附加值低的问题，使中间产品向终端产品、低端产品向高端产品扩展延伸。推动冶金、煤焦化、农产品加工等行业向精深加工转变，不断提高工业产品技术含量。开工建设永通球墨铸铁管铸造工业园、豫科龙盛空冷设备工业园等项目。完成利源集团年产30万吨煤焦油加氢项目主要设备安装，确保盈德气体合成气、飞翔电梯等项目竣工。加快企业结构转型。加强银企对接，帮助企业解决融资难题。继续组织开展产销对接、产用对接活动，促进产业链上下游对接。支持企业抱团取暖，建立产用联盟，通过实施战略重组做到"大而强"或者"小而精"、"小而专"，提升企业竞争力。加快产业发展载体建设。继续深化推进"四集一转"，统筹主导产业布局，加快公共服务平台建设，进一步提升产业集聚区综合带动效应，力争全年完成投资600亿元、规模以上工业主营业务收入超过1280亿元。新增标准化厂房90万平方米。

(二)着力强化新型城镇化引领，不断拓展发展空间。按照"1+4+2"城市组团式发展的总体布局，推进老城区、城市新区、新型农村社区联动协调，探索走好具有安阳特色的新型城镇化道路，推进产业集聚、人口集中、土地集约。增强新区辐射带动作用。突出"南扩东进"，加快安汤新城"两纵五横"路网、羑河综合治理及生态廊道建设，大力发展金融、商贸、高科技产业，促进产城融合。加快安东新城精密铸造、电子信息、物流等产业发展，形成沿京广高铁线布局的产业带。加强绿化水系等生态景观及人文景观建设，尽快促使安阳新区成规模、出形象、聚人气。积极推进洹北新区、新西区的规划建设。提升老城区综合承载能力。稳步推进旧城和"城中村"、棚户区改造。加强城市管理，继续开展城市环境综合整治，提升老城区服务功能和形象。完善城镇公共交通、生态环保、水电气热等基础设施和文化、卫生等公共服务设施，打造"文明、健康、和谐、有序、宜居"环境。推进城市组团发展。强化组团间内在联系和功能互补，促进交通衔接、产业链接、服务共享、生态共建，增强整体效应。加快中心城区与组团间快速通道建设，引导产业在中心城区与组团之间合理布局，实现分工协作、相互促进。增强城镇产业支撑能力，推动以产兴城，更好地解决进城农民就业问题，有序推进农业转移人口市民化。支持内黄县新型陶瓷、汤阴县食品医药、水冶钢铁、柏庄针织服装等产业快速发展。支持林州市、滑县发展成为功能配套的中等城市。完善综合交通体系。完成省道219线改造。开工建设西北绕城高速公路、省道301线升国道一级路改建工程、新郑机场安阳空港新城等项目。积极推进林桐高速公路、鹤辉高速公路林州段、安邯安鹤快速通道、晋豫鲁铁路和豫东北机场建设。加快城乡一体化。加快中心城区、县城、中心镇、新型农村社区协调发展，推进基础设施、公共服务向农村延伸，完善城乡发展一体化体制机制。按照"政策引领、规划先行、突出主体、保障权益、规范有序、拓展创新、互动联动、一体运作"的原则，完善规划、优化布局、积极稳妥、尽力而为，促进新型农村社区持续健康发展。

(三)着力做好"三农"工作，不断巩固农业基础地位。始终把解决好"三农"问题作为重中之重，全面落实强农惠农富农政策，加大投入，促进农业稳定发展、农民持续增收。实施高标准粮田"百千万"建设工程。推进粮食生产核心区建设，集合政策，整合资金，集中投入，实施粮食丰产科技工程，提高粮食综合生产能力，新建高标准粮田60万亩，力争粮食总产达到70亿斤。抓好南水北调配套工程、大中型灌区技改和病险水库除险加固、现代水利示范方建设。实施现代农业产业化集群培育工程。培育面品、瓜菜菌、油脂等十大农业产业化集群，新建扩建农业龙头企业20家，规范提升400家以上农民专业合作社。持续提升六大现代农业示范区建设水平，规划建设集成区10个。新增土地流转面积10万亩，温棚设施和立体高效农业9万亩。新建畜禽规模养殖场200个。改善农村生产生活条件。加快新一轮农网改造升级。解决25万人饮水安全问题。完成农村劳动力培训阳光工程1.3万人，贫困人口脱贫5.58万人。新增沼气用户1.5万户，大中型沼气工程50座，努力创建全国沼气示范市。

(四)着力深化开放招商和改革创新，不断培育竞争新优势。继续把对外开放作为应对复杂局面和破解各种制约的综合性战略举措，注重龙头基地型、技术创新型、服务中心型项目引进和签约项目落实。强化专业化集群式招商。制订专项行动方案，市县联动，重点在电子信息、食品医药、汽车及零配件、装备制造、新型建材、纺织等产业开展集群招商。办好第八届豫商大会，积极参加第八届中博会等重大招商活动，加强与世界500强、国内500强和央企深度战略合作，引进一批航空航材、高端制造、新兴服务业龙头企业，吸引中小企业配套跟进，形成雁阵效应。重点抓好与中航工业、国药集团、中建材、友嘉、海尔、万达、美的等企业合作项目的推进，积极引进直升机4S店、航空创意文化产业园区、航空港等项目。拓宽开放领域。推进工业领域开放的同时，加快服务业和社会事业领域的开放。引导外来资本和央企更多投向高成长性产业、战略性新兴产业、传统优势产业精深加工和现代农业，力争在金融保险、商贸物流、文化旅游等领域实现新突破。加大重点领域和关键环节改革力度。扎实做好国企改革扫尾攻坚工作。加强文化体制和文化市场综合执法改革。深化医药卫生、农村、财税、事业单位等改革。落实支持中小企业发展政策，鼓励促进非公有制经济发展。创新人才政策机制，引进发展急需的高层次、高技能人才。完善土地保障机制，优先支持重点项目、重点企业、产业集聚区、城市新区建设。加快构建自主创新体系。加快高新技术产业发展，积极创建全国科技进步先进市和国家知识产权示范市，新增省市企业技术中心、重点实验室等研发平台20个以上。充分发挥信息化的综合提升带动作用，加强信息化与"三化"融合发展，加快建设无线城市、智慧安阳、数字安阳。

(五)着力实施项目带动战略，不断夯实发展后劲。坚持项目建设不动摇，进一步整合资源，聚力推进，努力在项目规模和质量上实现新突破。深入实施项目带动战略。重点推进400个投资5000万元以上的重大项目建设，力争年底完成投资500亿元。抓好安钢高强度机械用钢、龙腾特钢高强度高性能棒线材、安化集团聚酯新材料等100个工业升级项目。加快实施安彩高科高端玻璃产业基地、大通无线通信产业园、三丰铜业高强高导电子引线框架材料等47个产业创新发展项目。抓好悠乐生活广场、五洲广场、欧凯龙家居广场等50个重大服务业项目。加快实施宇天化工煤焦油加氢、金钟新能源动力电池等80个工期短、水平高、投产快的项目。积极扩大投资。围绕国家资金投向和《中原经济区规划》支持重点，实施一批符合产业政策、带动能力强的大项目好项目，争取省、市重点项目达到200个。积极引导和启动民间资本投入到基础设施建设领域。优化项目建设环境。进一步完善重点项目周例会、市级领导分包、联审联批联办等制度，健全项目考核奖惩长效机制。组织开展全市重点项目观摩点评活动。力争新开工项目、竣工项目和年度投资目标100%完成。

(六)着力繁荣第三产业，不断打造经济发展新引擎。大力实施文化旅游发展战略，积极承接转移、促进集聚、打造品牌，带动第三产业发展提速、水平提升、比重提高。培育文化产业。推动文化产业转型升级，重点培育文化创意、影视制作等新兴文化产业，加快华强历史文化主题公园、周易文化产业园、红旗渠纪念馆、文化艺术中心等项目建设。继续推进殷墟国家考古遗址公园建设、曹操高陵保护开发利用和大运河安阳段申遗工作。积极推动三杨庄汉代遗址申报国家考古遗址公园。建设国际旅游城。重点打造中华第一都、太行大峡谷、汉字发源地、天河红旗渠等旅游品牌。积极推进十大文化旅游工程和"八个园"、"八个一"建设。组织好第五届中国·安阳国际航空运动旅游节、2013中国·安阳殷商文化旅游节等活动。加快发展低空经济和通用航空产业。力争全年接待游客增长9%以上，旅游收入增长9%以上。发展壮大服务业。加快商务中心区和特色商业区规划建设。开工建设中原(安阳)文化产业园、五洲工业博览城，扎实推进安阳物流港、众品冷链物流加工配送基地等规划建设。加快市场体系建设，推动商贸、金融、物流等现代服务业扩展规模。继续加大建筑业扶持力度，落实国家房地产调控政策，促进房地产业健康发展。

(七)着力改善生态环境，不断推进"美丽安阳"建设。全面落实节约资源和保护环境的基本国策，让安阳天蓝、水净、地绿，不断增强可持续发展能力。加强生态体系建设。推进林业"266"工程，营造林15.7万亩，新建提升都市林业观光园20个。加快生态廊道网络、农田防护林、城镇绿化建设，抓好京港澳高速安阳段绿化改造提升。积极创建国家生态园林城市、全国绿化模范城市。加大环境保护力度。严格环保准入和污染物排放标准，强化工程减排、结构减排、管理减排，加快燃煤电厂和水泥、钢铁企业脱硫脱硝等工程建设。深入开展安阳河、洪河、茶店河、羑河等综合治理，加强污水处理厂配套设施建设和运行管理。深化重点区域大气环境整治，加大市区扬尘、机动车尾气污染防治力度。积极开展连片综合整治，改善农村生产生活环境。加强资源能源节约。深入推进重点领域节能降耗，实施一批节能重点工程，推广节水灌溉和节水工艺技术、设备，降低资源能源消耗强度。大力发展循环经济，加快推进安西循环经济区建设，提高资源利用率。

(八)着力保障和改善民生，不断提升和谐安阳建设水平。认真办好"十项民生工程"，在学有所教、劳有所得、病有所医、老有所养、住有所居上下更大功夫，让安阳人民过上更好的生活。千方百计扩大就业。重点做好高校毕业生、城镇困难人员、退役军人就业工作。实现再就业3万人，其中就业困难群体再就业1万人，新增农村劳动力转移就业178万人。稳步增加居民收入。完善机关事业单位工资收入分配制度，健全职工工资正常增长机制和支付保障机制。增加城乡居民财产性收入，不断提高居民消费能力。抓好"米袋子"、"菜篮子"工程。强化物价管理，保持物价总水平基本稳定。积极推进社会保障体系建设。继续增加企业退休人员基本养老金，提高城乡低保、农村"五保"供养标准，提高优抚对象和困难群众补助水平。规范完善失业、医疗、工伤、生育保险市级统筹。完善减灾救灾、社会救助、社会福利制度，大力发展老龄、残疾人、红十字、慈善事业。加强保障性住房建设和管理，新开工保障性住房15101套。全面推进各项社会事业。继续实施学前教育三年行动计划，扩大学前教育资源。优化农村中小学布局，促进义务教育均衡发展。加大教育投入，鼓励引导社会力量办学。扎实推进文化惠民工程，着力完善市、县、乡、村四级公共文化服务网络。加强基层医疗卫生体系建设，提高基层卫生服务标准化水平，加快市公共卫生服务中心建设，促进基本公共卫生服务均等化。完善人口和计划生育公共服务体系，稳定低生育水平，提高出生人口素质。继续实施老年体育"长青工程"，广泛开展全民健身运动，提高竞技体育水平。加强和创新社会管理。畅通和规范群众诉求表达、利益协调、权益保障渠道，做好处置非法集资和信访工作。加强应急管理，提高突发事件应对处置能力。完善食品药品监管体制机制，强化安全生产监督，坚决遏制重特大事故发生。深入推进平安安阳建设，持续开展"扫黄打非"，依法打击违法犯罪活动，确保社会大局和谐稳定。

(九)着力转变职能，不断加强政府自身建设。认真落实中央关于改进工作作风、密切联系群众的八项规定，坚持"三具两基一抓手"工作方法、"四个明白"工作要求，深入推进"两转两提"，全面提升政府工作水平。提高行政效能。继续精简行政审批事项，加强政务服务体系建设，深化政务公开，为人民群众和市场主体提供优质高效服务。坚持关键在做，优化工作流程，完善工作机制，做到有部署、有督促、有奖惩，确保中央和省委、市委决策部署落到实处。加强政府绩效管理，以提高质量和效益为立足点，修订完善政府责任目标考核体系，引导转型升级。改进工作作风。完善干部直接联系群众制度，问政于民、问需于民、问计于民，办好顺民意、解民忧、惠民生的实事。坚持求真务实，勇于担当，重诺守信。改进调研方式，轻车简从、简化接待，多到情况复杂、意见较多、矛盾困难集中的地方，帮助解决问题。改进文风会风，减少检查评比，强化行政问责，真正把精力用到谋发展、抓落实上。严格依法行政。落实依法治国基本方略，严格按照法定权限和程序行使权力、履行职责、规范行为。自觉接受人大及其常委会的法律监督、工作监督和政协的民主监督，严格执行人大及其常委会的决议和决定，广泛听取各民主党派、工商联、无党派人士的意见和建议。强化行政复议能力建设，畅通行政复议渠道。加强公务员普法教育，提高依法行政能力。坚持从严治政。认真落实廉政建设责任制，完善惩防体系，加强监察、审计监督，规范权力运行，坚决惩处权力寻租、权钱交易等违法违规行为。厉行勤俭节约，反对铺张浪费，严控行政经费等一般性支出。深入创建学习型机关，开展公务员培训轮训，加强对城镇化、信息化、现代金融、现代物流、航空经济、社会管理等领域知识的学习，推进观念创新、工作创新、管理创新。加强和改进公务员管理，完善录用聘用、交流轮岗、竞争上岗等制度，健全创先争优长效机制，建设高素质公务员队伍。

各位代表!

时代赋予重任，奋斗铸就辉煌。再大的困难和挑战也阻挡不了我们共创美好明天的坚定步伐。我们坚信，2013年必定是安阳人民满怀豪情、奋发图强、披荆斩棘、开拓进取的一年。让我们更加紧密地团结在以习近平同志为总书记的党中央周围，在省委、省政府和市委的正确领导下，万众一心、振奋精神、扎实工作，为安阳人民生活得更加幸福、为建设中原经济区区域性中心强市而努力奋斗!
